# Ebola/Athens revisited.

**DOI:** 10.3201/eid0401.980127

**Published:** 1998

**Authors:** P E Olson, A S Benenson, E N Genovese


7. Luckhart S, Mullen GR, Wright JC. Etiologic agent of Lyme disease, *Borrelia burgdorferi*, detected in ticks (Acari: Ixodidae) collected at a focus in Alabama. J Med Entomol 1991;28:652-7.
8. Pung OJ, Banks CW, Jones DN, Krissinger MW. *Trypanosoma cruzi* in wild raccoons, opossums, and triatomine bugs in southeast Georgia, USA. J Parasitol 1995;81:324-6.
9. Lavender DR, Oliver JH Jr. Ticks (Acari:Ixodidae) in Bulloch County, Georgia. J Med Entomol 1996;33:224-31.
10. Spielman A, Wilson ML, Levine JF, Piesman J. Ecology of *Ixodes dammini* borne human babesiosis and Lyme disease. Annu Rev Entomol 1985;30:439-60.
11. Rich SM, Caporale DA, Telford SR III, Kocher TD, Hartl DL, Spielman A, et al. Distribution of the *Ixodes ricinus*-like ticks of eastern North America. Proc Natl Acad Sci U S A 1995;92:6284-8.
12. Norris DE, Klompen JSH, Keirans JE, Black WC IV. Population genetics of *Ixodes scapularis* (Acari:Ixodidae) based on mitochondrial 16S and 12S genes. J Med Entomol 1996;33:78-89.

### Ebola/Athens Revisited

**To the Editor:** After our hypothesis that the plague of Athens (430 B.C.–425 B.C.) could have been caused by Ebola virus was published in this journal (1996;2:155-6), it was brought to our attention that this hypothesis had been previously entertained.

Gayle D. Scarrow had published a paper entitled “The Athenian Plague: A Possible Diagnosis” in The Ancient History Bulletin 2.1 (1988). Unfortunately, this had not come to our attention in our literature search, and therefore we assumed that we were the first to recognize the possibility. Clearly, Ms. Scarrow deserves credit for suggesting this first. Her arguments are compelling, even without the support of more recently available information and the observations advanced in our publication.

We believe an evolving knowledge base (e.g., the information about the Côte d’Ivoire outbreak where a protracted epidemic has been meticulously documented) will serve to enhance the credibility of the Ebola/Athens hypothesis.

**P.E. Olson,* A.S. Benenson,† and E.N. Genovese†**
*U.S. Navy Balboa Hospital, San Diego, California, USA; †San Diego State University, San Diego, California, USA

